# The role of echocardiography for diagnosis and prognostic stratification in hypertrophic cardiomyopathy

**DOI:** 10.1007/s12574-020-00467-9

**Published:** 2020-04-16

**Authors:** Leonard Mandeş, Monica Roşca, Daniela Ciupercă, Bogdan A. Popescu

**Affiliations:** 1 University of Medicine and Pharmacy “Carol Davila”, Euroecolab, Bucharest, Romania; 2 Emergency Institute for Cardiovascular Diseases “Prof. Dr. C. C. Iliescu”, Şos. Fundeni 258, Sector 2, 022328 Bucharest, Romania

**Keywords:** Hypertrophic cardiomyopathy, Echocardiography, Diagnosis, Prognosis

## Abstract

Hypertrophic cardiomyopathy (HCM) is the most frequent cardiac disease with genetic substrate, affecting about 0.2–0.5% of the population. While most of the patients with HCM have a relatively good prognosis, some are at increased risk of adverse events. Identifying such patients at risk is important for optimal treatment and follow-up. While clinical and electrocardiographic information plays an important role, echocardiography remains the cornerstone in assessing patients with HCM. In this review, we discuss the role of echocardiography in diagnosing HCM, the key features that differentiate HCM from other diseases and the use of echocardiography for risk stratification in this setting (risk of sudden cardiac death, heart failure, atrial fibrillation and stroke). The use of modern echocardiographic techniques (deformation imaging, 3D echocardiography) refines the diagnosis and prognostic assessment of patients with HCM. The echocardiographic data need to be integrated with clinical data and other information, including cardiac magnetic resonance, especially in challenging cases or when there is incomplete information, for the optimal management of these patients.

## Introduction

Hypertrophic cardiomyopathy (HCM) is the most frequent disease with genetic substrate that involves the myocardium. The phenotype is usually heterogeneous as a result of both variability in the genetic mutations and incomplete penetrance in the affected population [1].

The current estimation of HCM prevalence (1 in 500 persons) is based on studies performed more than 20 years ago, notably the CARDIA cohort study [2–4]. Since the publication of those results, significant progress has been made in understanding the disease from both clinical and genetic perspectives, while the diagnostic tools have become more refined [5]. Thus, the true prevalence of HCM may be actually higher (up to 1 in 200 persons) [5–7]. While patients who are genotype positive–phenotype negative are not included in the prevalence estimates for HCM, they are nevertheless at increased risk of developing the disease, although the evolution to clinically significant disease is currently unpredictable [8–10].

Earlier diagnosis and proper prognostic stratification will allow reduction in disease-related morbidity/mortality by promoting timely treatment [11]. When first described, HCM was regarded as a rare disease affecting mostly the young, with a poor outcome, mainly related to the risk of sudden cardiac death (SCD) [12, 13]. Nowadays, it is recognized that HCM can affect patients of all ages and that the general prognosis of a patient with HCM is usually good, almost two-thirds having a normal life span with relatively low morbidity and with a general HCM-related mortality of about 0.7%/year [14–16]. However, some patients are at increased risk of SCD or of developing heart failure (HF)/atrial fibrillation (AF). Therefore, the identification of these patients is an important goal [1, 17]. Echocardiography is the cornerstone in screening, diagnosis, prognostic stratification and follow-up of HCM patients [1, 17, 18]. Echocardiographic measurements are included in current SCD risk calculators endorsed by the ESC and the AHA, respectively [1, 17]. Advanced echocardiographic techniques (tissue Doppler, two-dimensional speckle tracking) can help differentiate HCM from other causes of hypertrophy and identify patients at risk of SCD or of developing HF. Three-dimensional echocardiography offers more information regarding the distribution of hypertrophy, the LV mass, and the mechanism of dynamic LV obstruction [18].

## HCM diagnosis by echocardiography

Standard 2D echocardiography is the first-line imaging modality for the identification of LV hypertrophy (LVH) (Table 1). The current diagnostic criteria for HCM are an increase in LV wall thickness ≥ 15 mm in at least one myocardial segment or ≥ 13 mm for patients with a first-degree relative with confirmed HCM, in the absence of abnormal loading conditions/other causes of LVH (e.g., hypertension, valvular heart disease) [1, 17]. The measurement of LV wall thickness in parasternal short-axis views at end-diastole is the most accurate.

**Table 1:** Key echocardiographic features specific/suggestive for HCM [17–19, 25–27, 33–37]

	Echocardiographic parameter	Cutoff values suggesting HCM
Hypertrophy	Wall thickness / IVS to PW ratio	> 15 mm^a^, > 1.3^b^
	Distribution of hypertrophy	Asymmetric hypertrophy RV free wall hypertrophy ≥ 7 mm^c^ Reverse hypertrophic IVS
Mitral valve apparatus	Anterior leaflet elongation	AML > 30 mm (17 mm/m^2^)
	Posterior leaflet elongation	Absolute height of PL > 15 mm
	Papillary muscle abnormalities	Anterior displacement of AL PM
	Aorto-mitral angle	< 120°
	Mitral chordae	Elongation/thickening/buckling
	SAM	> 30% systolic contact with IVS
Systolic function	Systolic longitudinal dysfunction	Lateral S (TDI) < 4 cm/s Worse GLS (> − 10.6%)^d^ Paradoxical apical strain (apical HCM)
	Normal/supranormal radial strain	
Diastolic function^e^	Impaired relaxation	Lateral e’ < 4 cm/s
	Elevated filling pressures	Increase of A wave velocity during Valsalva maneuver^e^ LAVI > 34 mL/m^2 f^ Ar-A ≥ 30 ms E/e’ ratio > 10^g^ PAPs > 35 mmHg
Intraventricular obstruction	LVOT gradient /Midventricular obstruction	> 30 mmHg “Dagger shaped”/“Lobster claw” Doppler envelope

*HCM* hypertrophic cardiomyopathy, *IVS* interventricular septum, *PW* posterior wall, *RV* right ventricle, *AML* anterior mitral leaflet length, *PL* posterior leaflet, *AL PM* anterolateral papillary muscle, *SAM* systolic anterior motion, *TDI* tissue Doppler imaging, *Ar* duration of atrial reverse wave of the pulmonary venous flow, *A* duration of transmitral A wave, *PAPs* systolic pulmonary artery pressure

^a^Absence of abnormal loading conditions. 13 mm cutoff for HCM relatives

^b^1.5 for hypertensive patients

^c^Absence of abnormal loading conditions for the RV

^d^Reduction in longitudinal strain is greater for hypertrophied segments

^e^Diastolic dysfunction is the hallmark of the disease; filling pressures are elevated, even in the presence of an impaired relaxation pattern of the transmitral flow

^f^Absence of atrial fibrillation/significant mitral regurgitation

^g^Less specific in HCM as a surrogate for elevated filling pressures

**Fig. 1:**
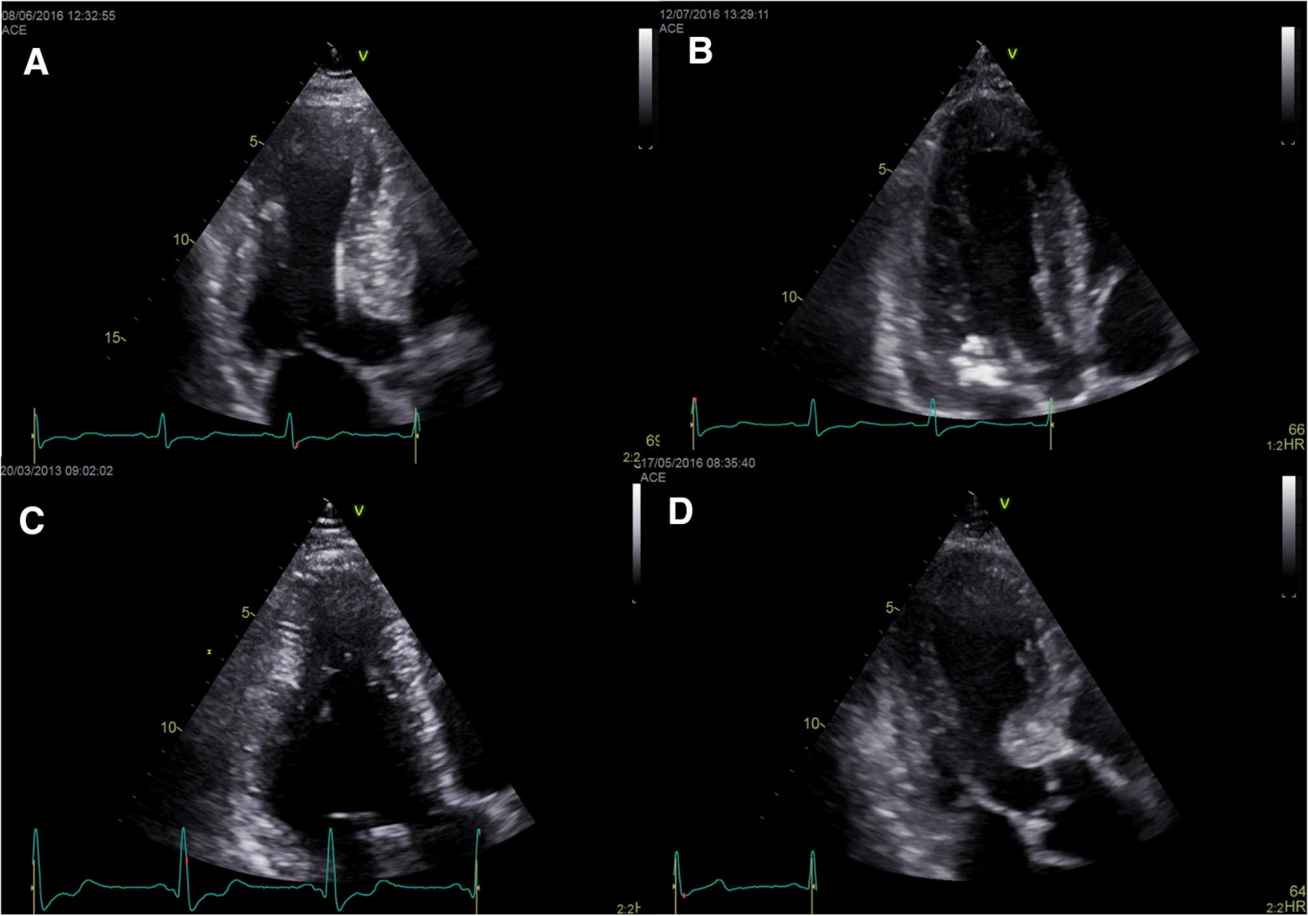
Various patterns of left ventricular hypertrophy that can be found in HCM patients. **a** Predominant asymmetric septal hypertrophy. **b** Concentric, symmetric hypertrophy. **c** Apical hypertrophy. **d** Isolated basal septal hypertrophy. *HCM* hypertrophic cardiomyopathy

While asymmetric hypertrophy (a septal-to-posterior wall thickness ratio ≥ 1.3 in normotensive patients or ≥ 1.5 in hypertensive patients) may be suggestive of HCM, it is not a specific finding (Fig. 1). Thus, about 10% of patients with hypertension (HTN) have asymmetric hypertrophy, and right ventricular (RV) hypertrophy can also lead to septal thickening [19]. Moreover, misalignment of the transducer beam can lead to oblique sections with wall thickness (WT) overestimation, while inclusion of RV structures (e.g., moderator band, trabeculations) when measuring the septum can also lead to a wrong HCM diagnosis [19]. The interventricular septum (IVS) morphology can also offer information about the presence of sarcomeric gene mutations. A reverse IVS curvature is associated with a high probability of disease-associated allele, while patients with a sigmoid IVS are much less likely to have a positive genetic test [20].

When native echocardiographic images are suboptimal, transpulmonary contrast echocardiography can improve visualization, especially when suspecting apical hypertrophy or an apical aneurysm [19].

The role of three-dimensional (3D) echocardiography is currently under discussion. It could improve the assessment of LV and LVOT geometry, and of LV mass.

Right ventricular hypertrophy is common, being found in more than 50% of the HCM patients, and it carries a worse prognosis. In the absence of secondary causes, it can act as an additional argument for HCM diagnosis. Caution should be taken not to include epicardial fat when measuring the RV free wall thickness [17, 19, 21].

Nevertheless, it should be noted that hypertrophy is a dynamic, noncontinuous and noncontiguous process in HCM, often affecting different, nonadjacent myocardial segments. It can be absent in childhood, appearing in adolescence/young adulthood and usually “stabilizing” with age. Wall thickness can also increase sharply later in life if there are additional causes for LVH (e.g., HTN, valvular heart disease), while patients with phenocopies or severe disease can have significant LVH from an early age [19, 22]. Some sarcomeric mutations (e.g., cardiac myosin binding protein C) lead to mild LVH, while carrying an increased risk for SCD [18].

## Other echocardiographic findings supporting the diagnosis of HCM

### Mitral valve apparatus abnormalities

While initially thought to be a disease limited to the myocardium, it is now well known that up to 59% of patients diagnosed with HCM have at least one abnormality of the mitral valve apparatus (MVA) as a direct effect of genetic mutations [23]. More commonly, leaflet elongation and excessive leaflet tissue are present in about 50% of patients, while other anomalies like chordal elongation, prolapse and direct insertion of the papillary muscle into the anterior leaflet are present in about 25% of cases [19]. The abnormalities also extend to the papillary muscles (PM) and may be related to their relative position (apical/anterior displacement), insertion (directly on the mitral valve) and number (duplication, bifidity)/hypertrophy [19].

Systolic anterior motion (SAM) of the mitral valve/chordae was once thought to be a very specific finding in HCM, being present in about 30–60% of the cases [24]. This theory is currently disproven, since other causes can also lead to SAM. These need to be taken into account when assessing the patient (e.g., severe HTN with small LV cavity treated aggressively, mitral valve surgical repair, severe hypovolemia, inotrope use) [18, 19].

Mitral regurgitation (MR) can be a result of MVA abnormalities, SAM (usually eccentric, posterior MR jet) and/or coexistence of mitral valve degenerative disease (usually central MR jet) [18, 19, 25].

### Left ventricular systolic function

Left ventricular ejection fraction (LVEF) is typically normal/supranormal in patients with HCM, and it only decreases in the late-stage “burnt-out” HCM in a small subset of patients (less than 15%) [17].

LVEF can remain normal in HCM because of the complex remodeling of LV structure and function. Thus, LVEF remains normal despite significant reduction in longitudinal and circumferential deformation, because of increased radial deformation in patients with increased WT and a small LV cavity [26]. Therefore, assessing myocardial deformation will better reflect LV systolic function in patients with HCM. Tissue Doppler imaging (TDI) can be used to assess mitral annular velocities and can detect subtle alteration in longitudinal function, even in segments without significant hypertrophy [27, 28]. While using TDI to assess strain and strain rate has Doppler-specific limitations (e.g., angle dependence), 2D-derived speckle tracking echocardiography (2D-STE) can provide more reproducible measurements of LV strain [29]. Typically, patients with HCM have a significant reduction in longitudinal strain (hypertrophied segments/segments with fibrosis being the most affected), even in early phases (subclinical systolic dysfunction), and a reduced LV untwisting [19, 29]. Moreover, paradoxical apical strain (systolic lengthening of apical segments) could be used to improve the diagnostic yield of echocardiography in apical HCM [30].

### Left ventricular diastolic function

One of the main mechanisms of HF in patients with HCM is LV diastolic dysfunction which occurs early in the disease evolution and is due to increased LV mass and stiffness [18, 19]. Transmitral flow is usually abnormal, and early diastolic myocardial velocity (e’) is frequently decreased, even in segments not affected by hypertrophy [31]. An increase in left atrium indexed volume (LAVI), especially if there is no significant MR/history of atrial fibrillation (AF), is a good surrogate for increased LV filling pressures [32]. It should be noted that transmitral flow E/A ratio and E/e’ ratio have poor/modest correlations with LV filing pressures in patients with HCM [33]. The Valsalva maneuver can be used in patients with an impaired relaxation pattern to unmask elevated LV filling pressures, proven by an increase in A wave velocity during the maneuver [34]. Table 1 summarizes the main echocardiographic parameters that can be used for assessing LV filling pressures [35]. In the presence of normal LAVI/LV filling pressures, the diagnosis of HCM is less likely, especially in elderly patients [18].

### Intraventricular obstruction in HCM

**Fig. 2:**
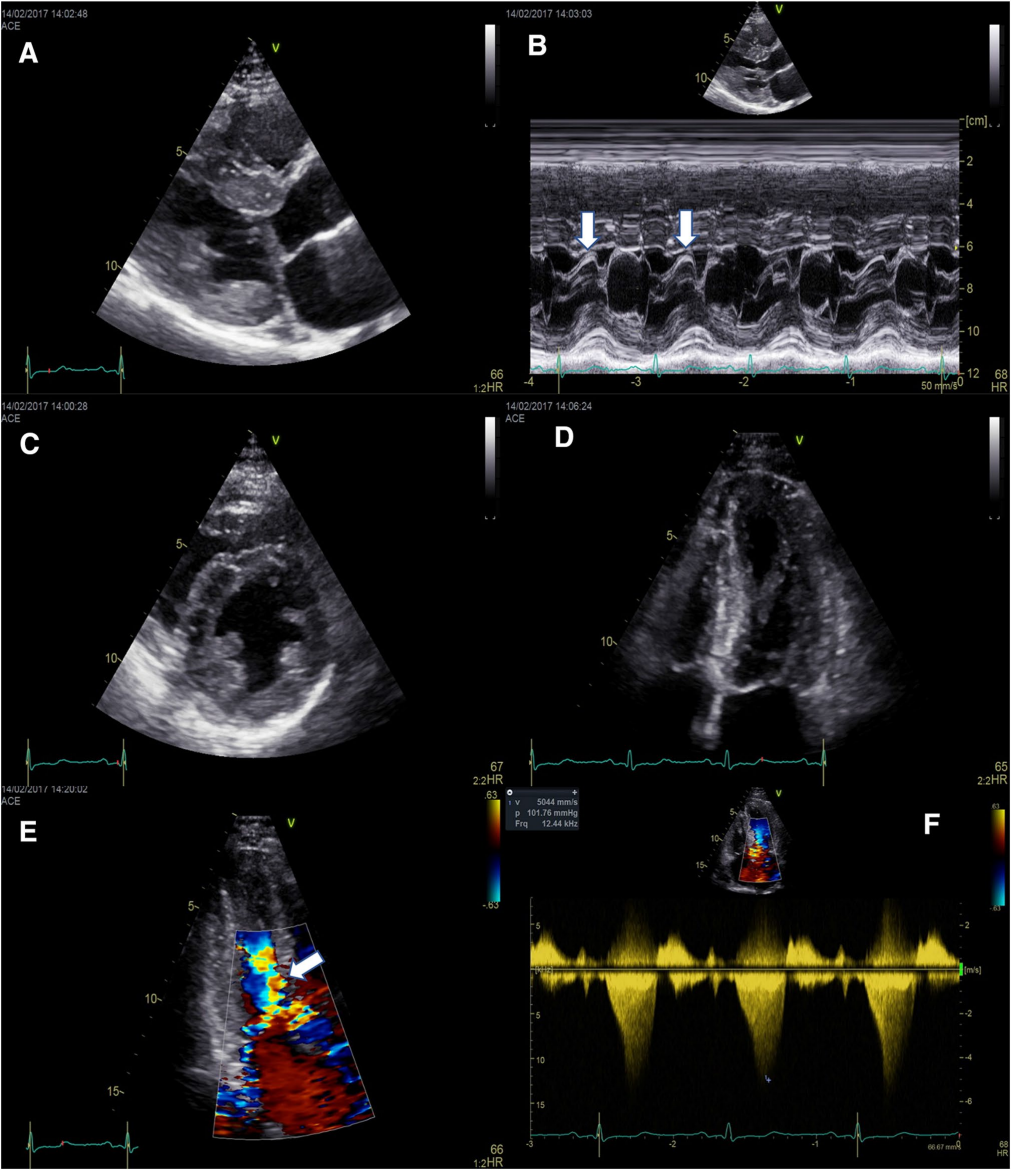
Complex mechanisms leading to dynamic obstruction in a patient with HCM. Concentric hypertrophy involving mainly the basal septum (diastolic IVS thickness of 15 mm), and elongated mitral leaflets with systolic anterior motion (**a**); M-mode echocardiography shows the systolic contact of the mitral valve with the IVS (arrows) (**b**); anterior displacement of the hypertrophied papillary muscles (**c**, **d**); moderate eccentric (posteriorly oriented) mitral regurgitation secondary to SAM (**e**); and significant resting LVOT obstruction by CW Doppler (peak resting gradient of 102 mmHg) (**f**). Of note, there is severe LVOT obstruction without severe septal hypertrophy, explained by the significant abnormalities of the mitral valve apparatus. *HCM* hypertrophic cardiomyopathy, *IVS* interventricular septum, *LVOT* left ventricular outflow tract

While usually located in the LVOT, the site of obstruction can also be midventricular. A peak gradient > 30 mmHg at rest or after provocative maneuvers (Valsalva/standing/exercise) is defined as intraventricular obstruction [17]. More than two-thirds of HCM patients have significant obstruction, but in half of them, this becomes apparent only after provocation [36]. Moreover, the intraventricular gradient has a significant variability, related to changes in loading conditions and in contractility [37]. In HCM, the main cause of LVOT obstruction is MVA abnormalities associated with a steeper LV to aortic root angle, leading to SAM, while the IVS thickness plays a lesser role by narrowing the LVOT (Fig. 2) [38]. Color flow mapping and pulse-wave (PW) Doppler can be used to identify the anatomic site of obstruction, and a careful assessment of the whole LV (apex/midventricular/LVOT) should be routinely made in all patients [18, 19]. Continuous-wave (CW) Doppler is useful in measuring the peak gradient. The Doppler envelope is typically “dagger shaped” (with an end-systolic peak), or like a “lobster claw” in cases of more severe obstruction (with a midsystolic temporary drop in pressure). Care should be taken not to measure the MR jet (which is “bell-shaped”), since this will overestimate obstruction severity [19]. Resting provocative maneuvers (e.g., Valsalva, standing) are mandatory in all patients [19].

Midventricular obstruction usually occurs in patients with significant midventricular hypertrophy and small LV cavity (“hourglass shaped ventricle”), more so if PM anomalies are present. It increases the risk of apical aneurysms that in turn predispose to ventricular arrhythmias and systemic embolism (in cases of apical thrombi) [18, 19].

Exercise echocardiography (EE, by treadmill/bicycle) is recommended in all symptomatic patients with resting intraventricular gradients < 50 mmHg or in asymptomatic patients when it is relevant for their medical treatment and for further risk stratification. Exercise echocardiography is a safe and feasible investigation [1]. Beside gradient provocation, exercise echocardiography is very useful for assessing exercise tolerance/symptoms, response to therapy, MR severity, blood pressure response, myocardial ischemia and exercise-induced arrhythmias [19, 39].

### Subclinical hypertrophic cardiomyopathy

Carriers of HCM gene mutations or subjects with ambiguous/negative genetic testing (30–40% of patients) who are asymptomatic and have some characteristics of HCM phenotype but do not fulfill the diagnostic criteria are considered to have subclinical HCM [40]. Even if the additional risk for SCD is very low in these patients, they should be carefully monitored with frequent echocardiograms, as opposed to HCM relatives with no abnormalities [41].

Echocardiographic findings include normal WT/borderline hypertrophy (12–14 mm), mitral valve leaflet elongation, myocardial crypts and myocardial apical trabeculations (the latter are better seen at cardiac magnetic resonance, CMR), while the LA is usually normal or only mildly dilated [19, 40]. TDI-derived myocardial velocities and 2D strain analysis can be useful since even patients with normal WT can have reduced myocardial velocities and mild segmental longitudinal dysfunction [42]. Moreover, exercise echocardiography can be performed to look for exercise-induced intraventricular gradients due to SAM, an additional finding suggestive of HCM [39].

### Advanced echocardiographic techniques

Three-dimensional echocardiography (3DE) has some advantages over standard 2D echocardiography. It can provide better information regarding the mechanism of intraventricular obstruction, distribution of hypertrophy, LV mass and systolic function. 3DE derived LV volumes, mass and ejection fraction have a better correlation with those obtained using CMR [19]. Moreover, 3DE can be useful for the differential diagnosis with other causes of LV hypertrophy. A novel index based on the standard deviation of the segmental mass volumes called the mass dispersion index (MDI) was proven to be significantly higher in patients with HCM, irrespective of the localization of hypertrophy [43, 44].

Dyssynchronous contraction in the absence of intra/interventricular conduction defects on the ECG is common in patients with HCM, especially if they have significant LVOT obstruction or septal hypertrophy [44, 45].

Key echocardiographic features for differentiating HCM from other diseases leading to LV hypertrophy are presented in Table 2.

**Table 2:** Echocardiographic features useful for differential diagnosis in HCM [18, 19, 49–52, 61–63]

Condition	Specific features (vs. HCM)
Athlete’s heart	Normal/slightly increased LV volumes
	Normal/mildly dilated LA
	Normal/supranormal annular systolic and diastolic velocities by TDI
	Normal GLS
	Reversible hypertrophy
Hypertensive heart disease	Symmetric hypertrophy^a^
	End-systolic SAM
	Mild to moderate systolic longitudinal dysfunction: better GLS (< − 10.6%)
	Reduced systolic radial strain
Cardiac amyloidosis	Concentric, biventricular hypertrophy
	Thickening of the interatrial septum/cardiac valves
	Hyperechoic walls (“speckled” appearance)
	Pericardial effusion
	Significantly decreased longitudinal strain/strain rate, with “apical sparing”
Fabry disease	Concentric, biventricular hypertrophy
	Thickening of the PM/cardiac valves
	Lateral LV wall is most often affected (reduced longitudinal strain)
	Circumferential strain is normal
Valvular/subvalvular obstruction	Concentric LV hypertrophy
	Valve calcifications/restricted leaflet mobility (valvular obstruction)
	Fibrous membrane/ring, discrete ridges or diffuse LVOT narrowing (subvalvular obstruction)
	Fixed LVOT obstruction with no SAM

*HCM* hypertrophic cardiomyopathy, *LV* left ventricle, *LA* left atrium, *TDI* tissue Doppler imaging, *GLS* global longitudinal strain, *PM* papillary muscles, *LVOT* left ventricular outflow tract, *SAM* systolic anterior motion of the mitral valve

^a^Asymmetric hypertrophy is uncommon (less than 10%)—when present, interventricular-to-posterior wall thickness ratio is < 1.3

## Prognostic stratification in patients with HCM

Echocardiography plays a central role in identifying markers associated with poor prognosis in patients with HCM (Table 3).

**Table 3:** Echocardiographic parameters with prognostic value in HCM [14, 17, 34, 40, 56–63]

Echocardiographic parameter	Value	Prognostic implication
Maximal WT	≥ 30 mm	3 × higher risk for VAs
LVOT obstruction	≥ 30 mmHg at rest, ≥ 50 mmHg (provoked)	Increased risk of SCD (1.5% vs. 0.9% per year)
		Increased risk of HF/HF progression^a^
		Increased risk of stroke
LA diameter	> 45 mm	Increased risk of SCD
		Increased risk of AF/AF recurrence
		Increased risk of stroke
LA volume^b^	≥ 37 mL/m^2^	Increased risk of AF
LA systolic strain^b^	≤ 23.4%	Increased risk of AF
		HF symptoms
Apical aneurysm	[≥ 4 cm]^c^	Increased risk of SCD (due to VAs and thrombus embolization)
RV hypertrophy	≥ 7 mm	Increased risk of VAs (NSVT)
		Increased risk of HF symptoms
Abnormal GLS	≥ − 16%	Increased risk of VAs
		Increased risk of HF/HF hospitalization/cardiac death
Systolic annular lateral wall velocity (S)	< 4 cm/s	Increased risk of HF/HF hospitalization
		Increased risk of cardiac death
Elevated filling pressures	E/e′ > 10, Ar-A ≥ 30 ms	Increased risk of HF/HF worsening
Mechanical dispersion	≥ 64 ± 22 ms	Increased risk of NSVT
		Correlates with fibrosis (LGE) at CMR

*WT* wall thickness, *VA* ventricular arrhythmias, *LVOT* left ventricular outflow tract, *SCD* sudden cardiac death, *HF* heart failure, *LA* left atrium, *AF* atrial fibrillation, *NSVT* nonsustained ventricular tachycardia, *GLS* global longitudinal strain, *LGE* late gadolinium enhancement, *CMR* cardiac magnetic resonance

^a^Patients with obstruction at rest have a higher risk than patients with provoked gradients (specific maneuvers/exercise echocardiography)

^b^Additional predictive value in patients considered at low risk for developing atrial fibrillation

^c^Significant increase in risk if apical aneurysm is larger than 4 cm

### Assessing the risk of sudden cardiac death

While in the community, most patients with HCM have a relatively good prognosis, with an estimated SCD risk of about 1% annually (compared to 0.2% in the general population), the risk can be significantly higher (over 6%/5 years) in patients presenting with more risk factors. Therefore, risk stratification is paramount to assess the need for an implantable cardioverter–defibrillator, which is the only effective treatment in reducing the SCD risk [1, 17].

Currently, there are two widely used multiparametric approaches for risk stratification, endorsed by ESC and AHA/ACC respectively, where all predictors are independently correlated with SCD. The risk model proposed by AHA/ACC is based on an international registry of 506 patients, has a very good sensitivity (SCD risk after applying the algorithm is less than 0.5%/year) and includes novel risk factors such as late gadolinium enhancement at CMR and the presence of LV apical aneurysms, beside maximal wall thickness > 30 mm, the presence of unexplained syncope, NSVT, family history of SCD and abnormal BP response to stress [17]. The main limitations are the fact that the model uses binary variables and does not include some risk modifiers [17]. The HCM SCD risk calculator proposed by the ESC is based on a multicenter cohort study of 3675 patients and provides individualized 5-year risk estimates using seven variables (age, maximum LV wall thickness, left atrial size, LVOT gradient at rest, family history of SCD, NSVT and unexplained syncope). Half of the parameters used in both models are derived from echocardiography [1].

The severity of hypertrophy assessed by maximal wall thickness (MWT) has been linked to VA, especially in younger patients. Patients with severe hypertrophy (MWT > 30 mm) have a threefold higher risk for VA. There are no data regarding the importance of ventricular mass/hypertrophy distribution and risk of VA [14, 45].

LVOT obstruction at rest increases the absolute risk of SCD from 0.9% to 1.5%. Unfortunately, the relation between severity of obstruction and SCD risk is less clear and there is low additive value in the absence of other risk factors. Moreover, LVOT gradients are highly variable [46].

LA remodeling is related to LV remodeling; therefore, an increase in LA diameter is a marker of disease severity. Currently, the relation between atrial fibrillation and SCD is still unclear. While the LA anteroposterior diameter is easy to measure, it is not ideal, since LA dilatation is not uniform, and LA diameter may underestimate the true LA size. Thus, LA volume may be a superior measure of LA size for risk stratification of patients with HCM [32]. However, more studies are needed to prove this hypothesis.

The presence of apical aneurysm increases the risk of SCD (40% patients have NSVT), with a significant prognostic value for aneurysms larger than 4 cm, and a mortality of 3.4%/year [47].

**Fig. 3:**
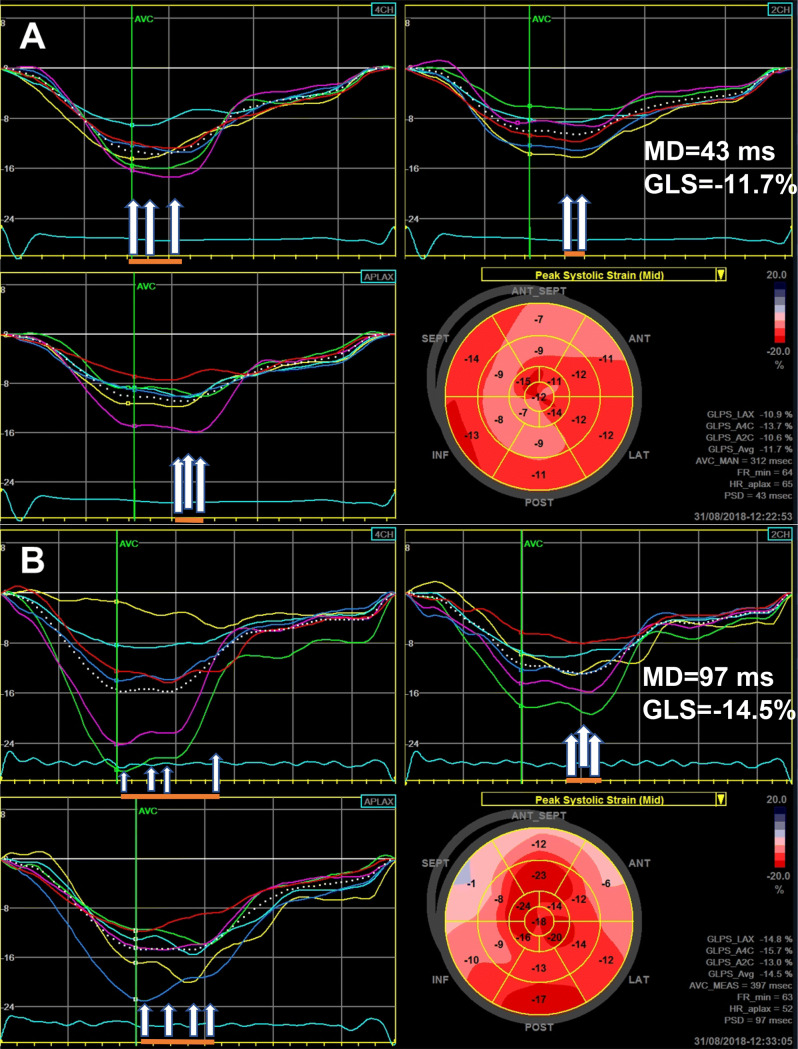
Global longitudinal strain (GLS) and myocardial dispersion (MD) in two patients with HCM. MD is calculated as standard deviation of the time from the beginning of ventricular systole to peak longitudinal shortening for each of the myocardial segments. The arrows mark the points of peak longitudinal shortening. A larger distance between arrows (orange line) means an increased MD. **a** Patient with HCM without history of VAs. **b** Patient with HCM and history of VAs (NSVTs) has a significantly higher MD. Moreover, patient B had significantly more fibrosis as assessed by CMR compared with patient A. Note that increased MD in patient B is independent of global longitudinal strain (which is better than for patient A), reflecting different functional information. *VA* ventricular arrhythmias, *HCM* hypertrophic cardiomyopathy, *NSVT* nonsustained ventricular arrhythmia, *CMR* cardiac magnetic resonance

Beside well-established echocardiographic risk factors, there are novel echo parameters derived from 2D STE analysis that may provide additional insight in SCD risk stratification. In a study on more than 3000 HCM patients, abnormal GLS was associated with VA [48]. Mechanical dispersion (i.e., the standard deviation of the time from the onset of systole to maximum contraction for each of the myocardial segments, an expression of heterogeneous contraction and electrical activation) relates to the extent of fibrosis (assessed by CMR) and is an independent predictor of VA (Fig. 3) [49].

### Assessing the risk of heart failure

The pathophysiology of HF in HCM is multifactorial—from diastolic dysfunction due to delayed LV relaxation, decreased chamber compliance, loss of LV suction and abnormal calcium homeostasis to LVOT obstruction and myocardial ischemia secondary to the reduction in myocardial blood supply, abnormal vasomotor response and vascular remodeling. Echocardiography plays an important role both in diagnosing cardiac remodeling and LVOT obstruction and in predicting progression to HF [19]. The finding of elevated LV filling pressure has a negative prognostic impact in HCM patients [50]. Right ventricular involvement, a common finding in about 50% of the patients, increases the risk for developing HF symptoms and VA (NSVT), with a direct correlation between RV wall thickness and heart failure symptoms [21].

Intraventricular obstruction increases the myocardial load and reduces the blood supply and cardiac output. The coexistence of significant MR can also worsen the HF symptoms. In symptomatic patients, in the absence of significant obstruction at rest (< 30 mmHg), gradient provocation (by specific maneuvers or by exercise echocardiography) should be pursued. LVOT obstruction is a predictor of HF symptoms (38% patients with resting obstruction have HF symptoms, compared with 20% of patients with provocable obstruction and 10% of patients without obstruction) and HF progression irrespective of its severity, with a greater magnitude in older patients (> 60 years) [51]. Exercise tolerance, lack of contractile reserve and the presence of inducible ischemia at exercise echocardiography have independent prognostic value [52].

Myocardial deformation can bring additional prognostic information. A marked reduction in LV systolic velocity by TDI (S < 4 cm/s at the lateral site) is an independent predictor of death or hospitalization for worsening HF [50]. Moreover, abnormal LV-GLS was associated with adverse composite cardiac outcomes [48]. Significant LA dysfunction (assessed by STE) has also been shown to correlate independently with HF symptoms [53].

### Assessing the risk for atrial fibrillation and the thromboembolic risk

**Fig. 4:**
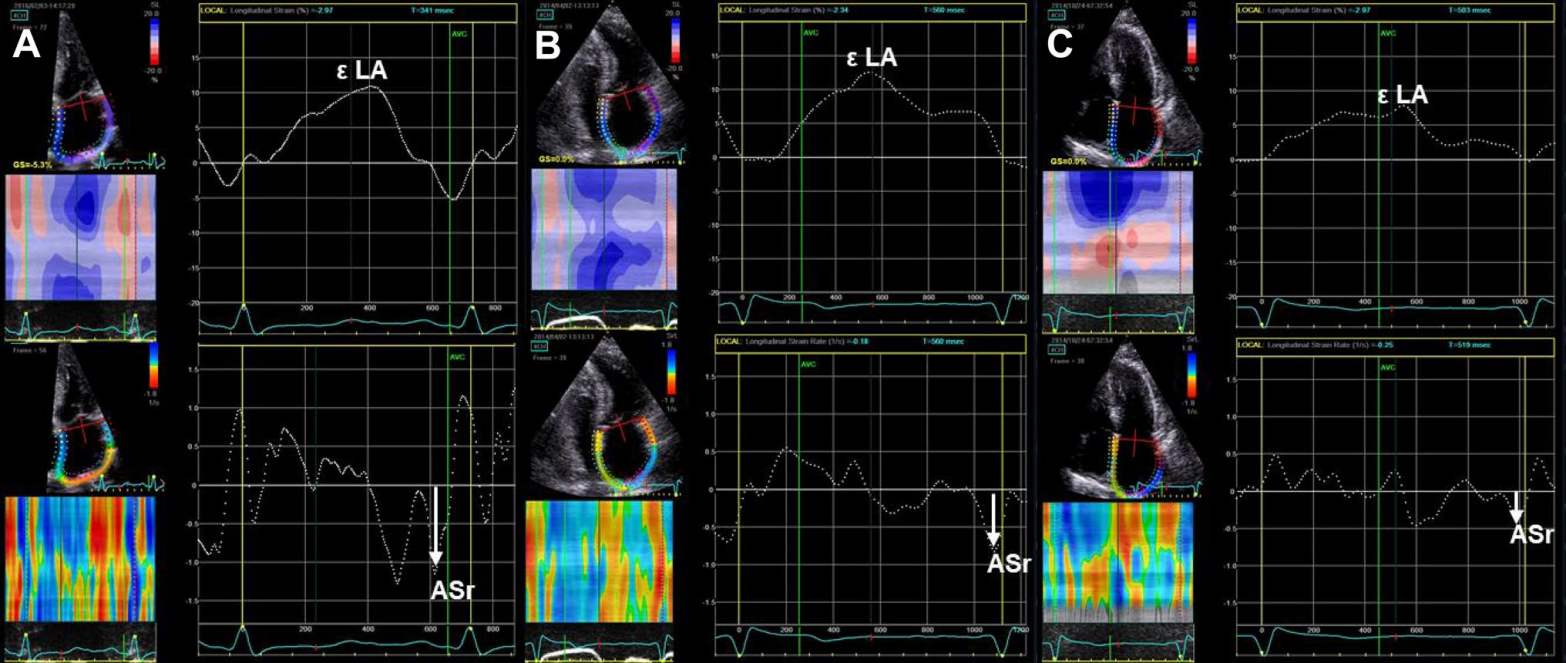
The assessment of LA function (reservoir function—ƐLA, and booster pump function—ASr) by speckle tracking echocardiography in three patients with HCM: **a** A patient with HCM, with anterior–posterior LA diameter < 45 mm, and without history of AF. **b** A patient with HCM, with anterior–posterior LA diameter < 45 mm, and with paroxysmal AF. **c** A patient with HCM, with anterior–posterior LA diameter > 45 mm, and with paroxysmal AF. Note that LA booster pump function is reduced in HCM patients in the presence of paroxysmal AF, furthermore if there is significant LA dilation (> 45 mm). *ƐLA* global longitudinal LA strain, *ASr* peak late diastolic strain rate, *LA* left atrium, *AF* atrial fibrillation, *HCM* hypertrophic cardiomyopathy, *DAS* anteroposterior LA diameter

The prevalence of AF in HCM is about 20–30%, compared to 1% in the general population [54]. The occurrence of AF increases morbidity and mortality leading to HF and thromboembolic events. Diagnosing AF can be difficult, since most paroxysmal AF episodes are asymptomatic. LA dimensions and age are independently associated with AF, but current tools to predict AF and thromboembolism lack in sensitivity and specificity [1, 17]. The ESC guidelines recommend screening for AF with ambulatory 48 h ECG monitoring for patients with an anteroposterior LA diameter > 45 mm [1]. Unfortunately, LA diameter lacks sensitivity. Among so-called low-risk patients (with an anteroposterior LA diameter < 45 mm), the incidence of AF is between 20 and 50% [54]. LA volume and strain can further refine risk stratification for AF in these patients, being more sensitive in detecting atrial remodeling, the main substrate of AF (Fig. 4). An indexed LA volume ≥ 37 mL/m^2^ and LA strain ≤ 23.4% were predictive for new-onset AF, with good accuracy (AUC = 0.83, and AUC = 0.78, respectively) [54]. Predictors for stroke include the presence of HF (NYHA class III/IV), age > 60 years, LVOT obstruction and AF/LA size > 45 mm (the most accurate predictors) [55].

## Limitations of echocardiography in HCM

While echocardiography is generally good in providing anatomical and functional details, it lacks the ability for tissue characterization. Cardiac magnetic resonance is very useful and may improve both the diagnosis and prognostic stratification in HCM, especially in patients with suboptimal echo images, borderline/conflicting echo findings or when suspecting phenocopies. It provides excellent morphological and functional data and information regarding the presence and distribution of myocardial fibrosis and extracellular volume [19].

## Conclusions

Echocardiography is an essential tool in patients with proven or suspected HCM. It provides important diagnostic information and allows detailed evaluation of LV systolic and diastolic function, presence and mechanism of LVOT obstruction, LA size and function. It also provides useful information for risk stratification (e.g., for SCD, heart failure, AF and stroke) to guide therapy. The echocardiographic data need to always be integrated with clinical data and other information, notably from CMR, especially in challenging cases, when there is conflicting information about the diagnosis or risk assessment.

## References

[1] Elliot PM, Anastasakis A, Borger MA (2014). 2014 ESC guidelines on diagnosis and management of hypertrophic cardiomyopathy: the task force for the diagnosis and management of hypertrophic cardiomyopathy of the European Society of Cardiology (ESC). Eur Heart J.

[2] Maron BJ, Gardin J, Flack JM (1995). Prevalence of hypertrophic cardiomyopathy in a general population of young adults. Echocardiographic analysis of 4111 subjects in the CARDIA study. Coronary artery risk development in (young) adults. Circulation.

[3] Hada Y, Sakamoto T, Amano K (1987). Prevalence of hypertrophic cardiomyopathy in a population of adult Japanese workers as detected by echocardiographic screening. Am J Cardiol.

[4] Maron BJ, Spirito P, Roman MJ (2004). Prevalence of hypertrophic cardiomyopathy in a population-based sample of American Indians aged 51 to 77 years (the Strong Heart Study). Am J Cardiol.

[5] Semsarian C, Ingles J, Maron MS (2015). New perspectives on the prevalence of hypertrophic cardiomyopathy. J Am Coll Cardiol.

[6] Seidman J, Seidman C (2001). The genetic basis for cardiomyopathy: from mutation identification to mechanistic paradigms. Cell.

[7] MacArthur D, Manolio T, Dimmock D (2014). Guidelines for investigating causality of sequence variants in human disease. Nature.

[8] Maron BJ, Yeates L, Semsarian C (2011). Clinical challenges of genotype positive (+)-phenotype negative (−) family members in hypertrophic cardiomyopathy. Am J Cardiol.

[9] Gray B, Ingles J, Semsarian C (2011). Natural history of genotype positive-phenotype negative patients with hypertrophic cardiomyopathy. Int J Cardiol.

[10] Maron BJ, Casey SA, Hauser RG (2003). Clinical course of hypertrophic cardiomyopathy with survival to advanced age. J Am Coll Cardiol.

[11] Rowin EJ, Maron MS, Casey SA (2013). Evidence for reduced mortality in an adult cohort with hypertrophic cardiomyopathy. Circulation.

[12] Hadarson T, De la Calzada CS, Curier R (1973). Prognosis and mortality of hypertrophic obstructive cardiomyopathy. Lancet.

[13] Shah PM, Adelman AG, Wigle ED (1974). The natural (and unnatural) history of hypertrophic obstructive cardiomyopathy. Circ Res.

[14] Spirito P, Autore C, Formisano F (2014). Risk of sudden death and outcome in patients with hypertrophic cardiomyopathy with benign presentation and without risk factors. Am J Cardiol.

[15] Maron BJ, Rowin EJ, Casey SA (2012). Risk stratification and outcome of patients with hypertrophic cardiomyopathy ≥ 60 years of age. Circulation.

[16] Rowin EJ, Hausvater A, Link MS (2017). Clinical profile and consequences of atrial fibrillation in hypertrophic cardiomyopathy. Circulation.

[17] Gersh BJ, Maron BJ, Bonow RO (2011). 2011 ACCF/AHA guideline for the diagnosis and treatment of hypertrophic cardiomyopathy. A report of the American College of Cardiology Foundation/American Heart Association Task Force on practice guidelines. Circulation.

[18] Williams LK, Frenneaux MP, Steeds RP (2009). Echocardiography in hypertrophic cardiomyopathy diagnosis, prognosis, and role in management. Eur J Echocardiogr.

[19] Cardim N, Galderisi M, Edvardsen T (2015). Role of multimodality cardiac imaging in the management of patients with hypertrophic cardiomyopathy: an expert consensus of the European Association of Cardiovascular Imaging Endorsed by the Saudi Heart Association. Eur Heart J Cardiovasc Imaging.

[20] Bos JM, Towbin JA, Ackerman MJ (2009). Diagnostic, prognostic, and therapeutic implications of genetic testing for hypertrophic cardiomyopathy. J Am Coll Cardiol.

[21] Rosca M, Calin A, Beladan CC (2015). Right ventricular remodeling, its correlates, and its clinical impact in hypertrophic cardiomyopathy. J Am Soc Echocardiogr.

[22] Maron MS, Maron BJ, Harrigan C (2009). Hypertrophic cardiomyopathy phenotype revisited after 50 years with cardiovascular magnetic resonance. J Am Coll Cardiol.

[23] Hoigne P, Attenhofer Jost CH, Duru F (2006). Simple criteria for differentiation of Fabry disease from amyloid heart disease and other causes of left ventricular hypertrophy. Int J Cardiol.

[24] Maron BJ, Epstein SE (1980). Hypertrophic cardiomyopathy. Recent observations regarding the specificity of three hallmarks of the disease: asymmetric septal hypertrophy, septal disorganization and systolic anterior motion of the anterior mitral leaflet. Am J Cardiol.

[25] Sun J, Yang X, Wang S (2017). The role of echocardiography in hypertrophic cardiomyopathy. CIVA.

[26] Stokke TM, Hasselberg NE, Smedsrud MK (2017). Geometry as a confounder when assessing ventricular systolic function: comparison between ejection fraction and strain. J Am Coll Cardiol.

[27] McMahon CJ, Nagueh SF, Pignatelli RH (2004). Characterization of left ventricular diastolic function by tissue Doppler imaging and clinical status in children with hypertrophic cardiomyopathy. Circulation.

[28] Bayrak F, Kahveci G, Mutlu B (2008). Tissue Doppler imaging to predict clinical course of patients with hypertrophic cardiomyopathy. Eur J Echocardiogr.

[29] Nagueh SF, McFalls J, Meyer D (2003). Tissue Doppler imaging predicts the development of hypertrophic cardiomyopathy in subjects with subclinical disease. Circulation.

[30] Reddy M, Thatai D, Bernal J (2008). Apical hypertrophic cardiomyopathy: potential utility of strain imaging. Eur J Echocardiogr.

[31] Severino S, Caso P, Galderisi M (1998). Use of pulsed Doppler tissue imaging to assess regional left ventricular diastolic dysfunction in hypertrophic cardiomyopathy. Am J Cardiol.

[32] Nistri S, Olivotto I, Betocchi S (2006). Prognostic significance of left atrial size in patients with hypertrophic cardiomyopathy (from the Italian Registry for Hypertrophic Cardiomyopathy). Am J Cardiol.

[33] Nishimura R, Appleton C, Redfield MM (1996). Noninvasive Doppler echocardiographic evaluation of left ventricular filling pressures in patients with cardiomyopathies: a simultaneous Doppler echocardiographic and cardiac catheterization study. J Am Coll Cardiol.

[34] Schwammenthal E, Popescu BA, Popescu AC (2000). Noninvasive assessment of left ventricular end-diastolic pressure by the response of the transmitral a-wave velocity to a standardized Valsalva maneuver. Am J Cardiol.

[35] Nagueh SF, Appleton CP, Gillebert TC (2009). Recommendations for the evaluation of left ventricular diastolic function by echocardiography. Eur J Echocardiogr.

[36] Maron MS, Olivotto I, Betocchi S (2003). Effect of left ventricular outflow tract obstruction on clinical outcome in hypertrophic cardiomyopathy. N Engl J Med.

[37] Kizilbash AM, Heinle SK, Grayburn PA (1998). Spontaneous variability of left ventricular outflow tract gradient in hypertrophic obstructive cardiomyopathy. Circulation.

[38] Levine RA, Vlahakes GJ, Lefebvre X (1995). Papillary muscle displacement causes systolic anterior motion of the mitral valve. Experimental validation and insights into the mechanism of subaortic obstruction. Circulation.

[39] Rowin EJ, Maron BJ, Olivotto I (2017). Role of exercise testing in hypertrophic cardiomyopathy. JACC Cardiovasc Imaging.

[40] Soler R, Mendez C, Rodriguez E (2018). Phenotypes of hypertrophic cardiomyopathy. An illustrative review of MRI findings. Insights Imaging.

[41] Efthimiadis GK, Pagourelias ED, Hadjimiltiades S (2015). Feasibility and significance of preclinical diagnosis in hypertrophic cardiomyopathy. Cardiol Rev.

[42] Yiu KH, Atsma DE, Delgado V (2012). Myocardial structural alteration and systolic dysfunction in preclinical hypertrophic cardiomyopathy mutation carriers. PLoS ONE.

[43] Caselli S, Pelliccia A, Maron MS (2008). Differentiation of hypertrophic cardiomyopathy from other forms of left ventricular hypertrophy by means of three-dimensional echocardiography. Am J Cardiol.

[44] D'Andrea A, Caso P, Severino S (2005). Association between intraventricular myocardial systolic dyssynchrony and ventricular arrhythmias in patients with hypertrophic cardiomyopathy. Echocardiogr J Card.

[45] Spirito P, Bellone P, Harris KM (2000). Magnitude of left ventricular hypertrophy and risk of sudden death in hypertrophic cardiomyopathy. N Engl J Med.

[46] Popescu BA, Rosca M, Schwammenthal E (2015). Dynamic obstruction in hypertrophic cardiomyopathy. Curr Opin Card.

[47] Rowin EJ, Maron BJ, Haas TS (2017). Hypertrophic cardiomyopathy with left ventricular apical aneurysm: implications for risk stratification and management. J Am Coll Cardiol.

[48] Tower-Rader A, Mohananey D, To A (2019). Prognostic value of global longitudinal strain in hypertrophic cardiomyopathy. A systematic review of existing literature. JACC Cardiovasc Imaging.

[49] Haland TF, Almaas VM, Hasselberg NE (2016). Strain echocardiography is related to fibrosis and ventricular arrhythmias in hypertrophic cardiomyopathy. Eur Heart J Cardiovasc Imaging.

[50] Kitaoka H, Kubo T, Okawa M (2011). Tissue doppler imaging and plasma BNP levels to assess the prognosis in patients with hypertrophic cardiomyopathy. J Am Soc Echocardiogr.

[51] Maron MS, Rowin EJ, Olivotto I (2016). Contemporary natural history and management of nonobstructive hypertrophic cardiomyopathy. J Am Coll Cardiol.

[52] Picano E, Ciampi Q, Citro R (2017). Stress echo 2020: the international stress echo study in ischemic and non-ischemic heart disease. Echocardiogr J Card.

[53] Rosca M, Popescu BA, Beladan CC (2010). Left atrial dysfunction as a correlate of heart failure. J Am Soc Echocardiogr.

[54] Debonnaire P, Joyce E, Hiemstra Y (2017). Left atrial size and function in hypertrophic cardiomyopathy patients and risk of new-onset atrial fibrillation. Circ Arrhythm Electrophysiol.

[55] Maron BJ, Olivotto I, Bellone P (2002). Clinical profile of stroke in 900 patients with hypertrophic cardiomyopathy. J Am Coll Cardiol.

[56] Chang SA, Kim HK, Lee SC (2013). Assessment of left ventricular mass in hypertrophic cardiomyopathy by real-time three-dimensional echocardiography using single-beat capture image. J Am Soc Echocardiogr.

[57] De Gregorio C, Recupero A, Grimaldi P (2006). Can transthoracic live 3-dimensional echocardiography improve the recognition of midventricular obliteration in hypertrophic obstructive cardiomyopathy?. J Am Soc Echocardiogr.

[58] Muraru D, Niero A, Rodriguez-Zanella H (2018). Three-dimensional speckle-tracking echocardiography: benefits and limitations of integrating myocardial mechanics with three-dimensional imaging. Cardiovasc Diagn Ther.

[59] Papadakis M, Carre F, Kervio G (2011). The prevalence, distribution, and clinical outcomes of electrocardiographic repolarization patterns in male athletes of African/Afro-Caribbean origin. Eur Heart J.

[60] Vinereanu D, Florescu N, Schulthorpe N (2011). Differentiation between pathologic and physiologic left ventricular hypertrophy by tissue Doppler assessment of long-axis function in patients with hypertrophic cardiomyopathy or systemic hypertension and in athletes. Am J Cardiol.

[61] Doi YL, McKenna WJ, Oakley CM (1983). ‘Pseudo’ systolic anterior motion in patients with hypertensive heart disease. Eur Heart J.

[62] Kato T, Noda A, Izawa H (2014). Discrimination of nonobstructive hypertrophic cardiomyopathy from hypertensive left ventricular hypertrophy on the basis of strain rate imaging by tissue Doppler ultrasonography. Criculation.

[63] Serra W, Marziliano N (2019). Role of cardiac imaging in Anderson–Fabry cardiomyopathy. J Cardiovasc Ultrasound.

